# Inclusion bodies: a new concept

**DOI:** 10.1186/1475-2859-9-80

**Published:** 2010-11-01

**Authors:** Elena García-Fruitós

**Affiliations:** 1 CIBER en Bioingeniería, Biomateriales y Nanomedicina (CIBER-BBN), Bellaterra, 08193 Barcelona, Spain; 2 Institut de Biotecnologia i de Biomedicina and Departament de Genètica i de Microbiologia, Universitat Autònoma de Barcelona, 08193 Bellaterra (Cerdanyola del Vallès), Barcelona, Spain

## Abstract

**In the last decades, the understanding of inclusion body biology and consequently, of their properties and potential biotechnological applications have dramatically changed. Therefore, the development of new purification protocols aimed to preserve those properties is becoming a pushing demand**.

## Commentary

The formation of inclusion bodies (IBs) in bacteria, especially in *Escherichia coli*, has become one of the most common undesirable events when using these microbial cell factories to produce soluble recombinant proteins for both research and industrial applications [1-3]. Since IBs have been long considered a waste product, essentially formed by misfolded proteins, prone to aggregate, important efforts have been made to eliminate, or at least partially reduce, its formation [4]. Gene dosage, promoter strength, mRNA stability, codon usage, culture temperature, protein and genetic engineering and overexpression of folding modulators are just some examples of a vast list of parameters that have been modulated to avoid IB formation [4].

However, based on publications listed on PubMed citation database in the last years, it is worth noting that the scene has completely changed. The first evidence of change was published in Microbial Cell Factories in 2005, when our group showed that IBs, contrarily to what had been widely believed, were particulate aggregates formed, at least partially, by biologically active polypeptides [5]. Since then, an avalanche of proofs that undoubtedly support this observation has been incessantly published. Apart from our publications [6-13], de Groot el al. [14,15] and Peternel el al. [16,17] have shown that Green Fluorescent Protein (GFP) embedded in such aggregates is highly fluorescent, when observed by both confocal microscopy and fluorescence spectroscopy. Jevsevar and collaborators have also recently proved that the cytokine human granulocyte colony-stimulating factor (hG-CSF) in IBs adopt a native structure, being consequently fully active [18]. Although the amount of evidences compromising the dogma describing IBs as inert aggregates, there is still an important question to be answered: why do not previous research data support these observations? The answer is simple: nobody checked IB functionality before (except Worall and Goss and Tokatlidis and collaborators, who described the presence of biologically active IBs [19,20], although they have for long leaved aside). In fact, the use of GFP as a reporter to study IB kinetics through fluorescence microscopy raised the alarm in 2005 [5]. Thereby, not long ago, the information concerning conformational quality of these proteins aggregates was simply neglected, being not information available with regard to this phenomenon.

When reviewing recent reports in which enzyme-based IBs are tested, it is observed that, in agreement with the observations done with GFP and other proteins, enzymes embedded in such aggregates are also active [5,7,21-26]. This observation not only corroborates the model regarding IB composition [27], but also opens a promising market in biocatalysis industry. In this context, it is worth highlighting that Nahálka and coworkers have made an important contribution to this field, as they have evaluated a wide number of examples [21-25,28]. Enzymes like galactosidases [5,7], reductases [5], oxidases [22,28], kinases [21,24], phosphorylases [25] and aldolases [23] have been produced as IBs and used to successfully catalyze specific reactions. Furthermore it should also be underlined that IBs can be easily removed from the reaction mixture by a simple centrifugation, allowing then the possibility to reuse them in other reaction cycles [21,23,25]. Therefore, based on these published studies and considering that the high cost of the catalyst production, the mass transfer problems occurring during the catalysis reaction and the lost of activity due to enzyme immobilization processes are important drawbacks in biocatalysis processes, the incorporation of IBs can draw an innovative and encouraging stage in the industrial catalysis market [29].

Focusing our attention on the search of other reported applications of IBs, we come up with a paper published in 2009 by Nahálka and collaborators [30]. In this work the authors explore the possibility of using IBs with lectin activity in a hemaaglutination assay in order to design a cheap diagnostic tool for the characterization of glycoproteins. IBs show to be once again a good alternative, in this case to the expensive and cumbersome lectin microarrays, being able to be used for the design of an array of glycoproteins.

Moreover, recent results show that IBs, as proteinaceous nanoparticles, could also open the doors of an encouraging future in regenerative medicine field, being these particulate aggregates a promising alternative [9-11] to the classical materials used for tissue engineering purposes [31-33].

It seems undeniable that IB concept has completely changed and, consequently, it is also becoming obvious that the existing protocols to purify these aggregates need to be adapted to this new reality. Up to now, since IBs have essentially been used for protein refolding attempts [34], protocols established to purify these aggregates had not important requirements. However, the possibility to use IBs as biocatalysts, as diagnostic tools or as biomaterials for tissue engineering, necessarily involve a careful redesign of the purification protocol strategy used.

Several publications show that both mechanical and non-mechanical lysis methods are equally used to break cell in order to obtain recombinant soluble proteins [35-37]. After cell disruption, the soluble fraction can be easily separated from cell debris by centrifugation, thus being the protein of interest finally isolated from the obtained supernatant by conventional purification procedures. On the other hand, even though both mechanical and non-mechanical cell disruption processes are also used to recover IBs, in this case it is necessary to take the insoluble fraction after the corresponding centrifugation step, which is generally washed, using detergents and/or DNase treatments, being the obtained product ready to be use for protein refolding purposes [38]. Nevertheless, given that an important amount of impurities such as membranes, membrane bound proteins, cell wall fragments, DNA, RNA and, even, viable cells are still present in the IB mixture, under these conditions these nanoparticles are not suitable for biotechnological and biomedical purposes. Consequently, the optimization of the IB purification strategy has come out as a new necessity and, in fact, in just few months a couple of papers discussing this issue have been published in Microbial Cell Factories [38,39]. Both publications point out that it is crucial to redefine an appropriate method to isolate highly pure IBs from bacterial cells, especially emphasizing the importance of choosing a suitable cell lysis strategy to completely eliminate the presence of any viable bacteria in the final sample, since this would be not acceptable when aiming to use such aggregates for the applications listed above. Even though the importance of having cell-free IBs, it is also critical to bear in mind that the isolation process should not damage neither IB structure nor the quality of the protein embedded inside these protein aggregates. Thus, the pursued objective is the establishment of a new protocol that does not compromise the applicability of IBs, neither for the presence of viable bacteria or impurities nor for the damage of the final product. Our group and Peternel and Komel have explored the effectiveness of lysis methods such as enzymatic lysis, sonication, freeze-thawing cycles and high-pressure homogenization, among others [38,39]. The obtained results show that non-mechanical lysis is gentle toward IB integrity, but not effective enough regarding cell disruption [39]. On the other hand, while cell lysis improve when using mechanical methods [38,39], the quality of protein trapped in IBs is frequently compromised [39]. Therefore, both groups agree that there is no method good enough to completely break bacterial cell wall, without damaging IB quality. Therefore, it could be concluded that the combination of both mechanical and non-mechanical lysis procedures could be a suitable election. Moreover, the published data from both groups in 2010 in Microbial Cell Factories also underline the existing variability among different strains overproducing different recombinant proteins, being some of them more sensitive to the cell lysis process than others [38,39], an effect that could be connected with the influence of the recombinant protein production on the membrane composition and permeability [40]. Altogether, these data point out that further studies are needed to approach an universal alternative to obtain pure and functional IB with preserved morphology, regardless of the recombinant protein. The exploration of an improved strategy should take into account not only the election of an appropriate lysis method, but also the design of the necessary washing steps to isolate native, undisturbed active protein nanoparticles.

Thus, it seems that a new age of IBs has just started, which should necessarily go accompanied by an optimization of the IB isolation protocol. In this line, it is important to note that such a procedure protocol has to be meticulously redesigned, analyzing point-by-point the spectrum of effects of any purification step in the final product.

## Competing interests

The authors declare that they have no competing interests.
